# Supplementary material for: Iterative improvement in the automatic modular design of robot swarms
Source: PeerJ Comput Sci. 2020 Dec 7;6:e322. doi: 10.7717/peerj-cs.322 (PMC7924708; doi:10.7717/peerj-cs.322)
Supplement: Supplemental Information 3 [file peerj-cs-06-322-s003.zip › argos3/doc/api/standalone/a00413_source.html]

ARGoS: plugins/robots/e-puck/simulator/dynamics2d\_epuck\_model.h Source File


- Main Page
- Related Pages
- Namespaces
- Classes
- Files

- File List
- File Members

# plugins/robots/e-puck/simulator/dynamics2d\_epuck\_model.h

Go to the documentation of this file.

```
00001 
00007 #ifndef DYNAMICS2D_EPUCK_MODEL_H
00008 #define DYNAMICS2D_EPUCK_MODEL_H
00009 
00010 namespace argos {
00011    class CDynamics2DDifferentialSteeringControl;
00012    class CDynamics2DGripper;
00013    class CDynamics2DGrippable;
00014    class CDynamics2DEPuckModel;
00015 }
00016 
00017 #include <argos3/plugins/simulator/physics_engines/dynamics2d/dynamics2d_single_body_object_model.h>
00018 #include <argos3/plugins/simulator/physics_engines/dynamics2d/dynamics2d_differentialsteering_control.h>
00019 #include <argos3/plugins/robots/e-puck/simulator/epuck_entity.h>
00020 
00021 namespace argos {
00022 
00023    class CDynamics2DEPuckModel : public CDynamics2DSingleBodyObjectModel {
00024 
00025    public:
00026 
00027       CDynamics2DEPuckModel(CDynamics2DEngine& c_engine,
00028                               CEPuckEntity& c_entity);
00029       virtual ~CDynamics2DEPuckModel();
00030 
00031       virtual void Reset();
00032 
00033       virtual void UpdateFromEntityStatus();
00034       
00035    private:
00036 
00037       CEPuckEntity& m_cEPuckEntity;
00038       CWheeledEntity& m_cWheeledEntity;
00039 
00040       CDynamics2DDifferentialSteeringControl m_cDiffSteering;
00041 
00042       const Real* m_fCurrentWheelVelocity;
00043 
00044    };
00045 
00046 }
00047 
00048 #endif
```

---

Generated on 10 Jul 2018 for ARGoS by 
 1.6.1
